# The influence of object shape and center of mass on grasp and gaze

**DOI:** 10.3389/fpsyg.2015.01537

**Published:** 2015-10-16

**Authors:** Loni Desanghere, Jonathan J. Marotta

**Affiliations:** ^1^Perception and Action Laboratory, Department of Psychology, University of Manitoba, WinnipegMB, Canada; ^2^Postgraduate Medical Education, College of Medicine, University of Saskatchewan, SaskatoonSK, Canada

**Keywords:** visuomotor control, fixations, gaze locations, grasp locations, irregular non-symmetrical objects

## Abstract

Recent experiments examining where participants look when grasping an object found that fixations favor the eventual index finger landing position on the object. Even though the act of picking up an object must involve complex high-level computations such as the visual analysis of object contours, surface properties, knowledge of an object’s function and center of mass (COM) location, these investigations have generally used simple symmetrical objects – where COM and horizontal midline overlap. Less research has been aimed at looking at how variations in object properties, such as differences in curvature and changes in COM location, affect visual and motor control. The purpose of this study was to examine grasp and fixation locations when grasping objects whose COM was positioned to the left or right of the objects horizontal midline (Experiment 1) and objects whose COM was moved progressively further from the midline of the objects based on the alteration of the object’s shape (Experiment 2). Results from Experiment 1 showed that object COM position influenced fixation locations and grasp locations differently, with fixations not as tightly linked to index finger grasp locations as was previously reported with symmetrical objects. Fixation positions were also found to be more central on the non-symmetrical objects. This difference in gaze position may provide a more holistic view, which would allow both index finger and thumb positions to be monitored while grasping. Finally, manipulations of COM distance (Experiment 2) exerted marked effects on the visual analysis of the objects when compared to its influence on grasp locations, with fixation locations more sensitive to these manipulations. Together, these findings demonstrate how object features differentially influence gaze vs. grasp positions during object interaction.

## Introduction

We move about and interact with objects in our environment so effortlessly that the complexities of these interactions are rarely noticed. Although the integration of various senses, such as visual and tactile feedback when locating and picking up objects and vestibular information for balance (for review see Kandel et al., 2000), plays key roles in our interactions, we primarily rely on our sense of vision to accurately carry out our movements, with eye movements typically preceding hand movements in both pointing (Abrams et al., 1990; Bekkering et al., 1994; van Donkelaar et al., 2004) and object manipulation tasks (Land et al., 1999; Johansson et al., 2001; Land and Hayhoe, 2001; Hayhoe et al., 2003; Hayhoe and Ballard, 2005). When you want to pick up an object, it is usually a simple matter to look where you remember leaving it, reach out to its location, and accurately pick it up. However, we do not typically grasp objects in arbitrary locations or look at random parts of that object during these actions. Instead, how we interact with an object depends on both the ongoing and planned behavior of the individual (Hamed et al., 2002) and the cognitive demands of the task (Yarbus, 1967).

For example, where we look on an object will vary depending on whether we are directly interacting with that object (e.g., picking it up; Johansson et al., 2001; de Grave et al., 2008; Brouwer et al., 2009; Desanghere and Marotta, 2011; Prime and Marotta, 2013), performing a series of movements (e.g., making sandwiches or tea; Ballard et al., 1992; Smeets et al., 1996; Land et al., 1999; Hayhoe et al., 2003), or completing a perceptual task such as visual search (Findlay, 1997; Zelinsky et al., 1997; Klein, 2000; Araujo et al., 2001), viewing objects (He and Kowler, 1991; Kowler and Blaser, 1995; McGowan et al., 1998; Melcher and Kowler, 1999; Vishwanath et al., 2000; Vishwanath and Kowler, 2003, 2004), or size estimation (Desanghere and Marotta, 2011). Indeed, visuomotor control is a complex, interactive process between perception and action; reflective in the separate yet interconnected neural pathways dedicated to these processes (for review see Milner and Goodale, 1995; Goodale, 1998, 2014; Schenk and McIntosh, 2010). Not only do we use vision to identify objects with which we are interacting, vision also provides us with feedback about the approaching hand (toward the object) to enable online corrections (Binsted et al., 2001; Riek et al., 2003), as well as provides information about where the contact location on the object is relative to the arm’s motor system (Land and Hayhoe, 2001; Soechting et al., 2001). Research has shown that eye movements are typically initiated toward the object 40–100 ms prior to movement onset (Prablanc et al., 1979; Biguer et al., 1982; Land et al., 1999) with fixations linked to where participants grasp an object (i.e., they look at the location where they place their index finger during a precision grasp; Brouwer et al., 2009; Desanghere and Marotta, 2011; Prime and Marotta, 2013), and, when manipulating the objects, linked to forthcoming grasp sites, obstacles, and landing sites where objects are subsequently grasped, moved around, and placed, respectively (Johansson et al., 2001).

How fixations change throughout a reach-to-grasp movement and exactly what object properties are fixated has yet to be fully explored. A growing body of research has investigated *where* on an object people are fixating during basic reaching and grasping movements to simple objects. For example, de Grave et al. (2008) examined fixation locations during a reaching and grasping task to objects that were either fully visible or that had the index finger, thumb, or both grasp locations partially occluded. They found that first and second fixations on the objects were above the object’s center of mass (COM), as well as above the visible COM (calculation of the COM based on the visible surface area of the object) in the case of partly occluded objects. In both instances fixation locations were toward index finger grasp location. However, similar to Johansson et al. (2001) where participants were instructed to grasp the object at a specific location, participants in this experiment also had specific grasp locations where they had to place their index finger and thumb on relatively simple objects such as a triangle or cross. In a later investigation using similar objects, Brouwer et al. (2009) contrasted fixation locations when participants were reaching out to grasp an object vs. when they were asked to simply view that object. They found that during first fixations to the objects there was no difference between tasks; during both grasping and viewing participants were looking closer to the COM. During second fixations, however, fixations while grasping were found to be significantly higher up on the objects (toward index finger location) than those found during viewing. Desanghere and Marotta (2011) and later Prime and Marotta (2013) showed the opposite pattern in fixations when grasping simple symmetrical objects (squares and rectangles), with fixation locations first directed towards the grasp site for the index finger, and then directed lower toward the object’s COM just prior to contact with the object. In these experiments, participant’s grasp axis (the imaginary line joining the contact points of the index finger and thumb on the object) coincided with the object’s COM location.

These studies all suggest that where we look on an object plays a key role in real-time grasping movements, with grasp and gaze locations sensitive to COM location and linked to the eventual index finger grasp location. In everyday life; however, we are not always reaching out to grasp simple symmetrical objects. Our visuomotor system must deal with complex calculations such as surface curvature and differences in an object’s COM. Indeed, the role of vision in grasping is not only to activate appropriate grasp schemas, but also to determine accurate positioning of the fingers on the objects (Jeannerod et al., 1995; Smeets and Brenner, 1999). However, it is not always a simple relationship between fixation location and focal attention (i.e., what someone is concentrating on), as visual-spatial attention can be directed either overtly by actively fixating the eyes (the fovea) onto a specific area or covertly, by allocating cognitive resources to process information that is located in another region of space (Posner, 1980; Irwin, 2004). In this way, we are able to successfully reach out and pick up objects outside of foveal vision. Despite this; however, research has shown that when reaching out to pick up objects (Brouwer et al., 2009; Desanghere and Marotta, 2011; Prime and Marotta, 2013), participants’ fixation locations do not stray off the object and are linked to the most relevant dimensions of the objects (index finger grasp location during a precision grasp), even in instances where digit trajectories of the thumb are made more variable (Cavina-Pratesi and Hesse, 2013). In other words, despite participants’ ability to fixate any part of the desired object or any part of the scene in front of them and still complete the task, participants in these experiments reliably fixate the eventual index finger grasp location on the object with which they are interacting.

As we can see, the relationship between index finger grasp location and fixation locations are well documented, demonstrating a reliable relationship between grasp locations and focal attention. However, the effects that object complexity and changes in COM may have on eye-hand coordination are under-explored. Although it has been shown that alterations in object shape affect various reaching and grasping kinematics such as minimum grip force (Jenmalm et al., 1998), maximum grip aperture (Eloka and Franz, 2011), and digit placement (Jeannerod, 1988; Goodale et al., 1994; Lederman and Wing, 2003; Marotta et al., 2003; Kleinholdermann et al., 2007), the relationship between where we look relative to where we grasp on irregular objects remains unclear. Given that current research suggests that both the relevant dimension of an object and the overall shape of the object facilitate ongoing movements during reaching and grasping tasks (Eloka and Franz, 2011), further investigations between eye-hand coordination when grasping more complex objects needs to be carried out.

The primary goal of this research was to examine the variability in fixation locations across several conditions on irregular shaped objects and explore the relationship of these fixation locations to grasp locations on those same objects. In Experiment 1, we investigated fixation and grasp locations to contoured objects that had an asymmetrical design and compared this behavior to grasps made to symmetrical objects (squares and rectangles). Both object types had identical maximum horizontal and vertical dimensions. In Experiment 2, we explored the effects of object shape and changes in COM location on fixation and grasp locations. Based on the literature it was expected that participant’s grasp axis and fixation locations would be shifted away from the horizontal center of the objects, toward the object’s COM (Experiments 1 and 2), and this distance would increase with increases in COM locations from that point (Experiment 2), and in both instances be linked to the eventual index finger grasp location. If object irregularity is affecting visual analysis of the object, we expect to see differences in fixation locations relative to symmetrical objects (Experiment 1) and grasp locations (Experiments 1 and 2). If fixation locations are identical across conditions (e.g., linked to index finger location and not influenced by object irregularity) this would suggest that the visual analysis of irregular shaped objects during object manipulation is taking in only the most relevant dimensions of the objects needed for grasping.

## Experiment 1

### Materials and Methods

#### Participants

Fourteen undergraduate psychology students (nine female) between the ages of 18 and 30 (*M* = 21 years-old) were recruited for participation in this study. All participants were shown to be strongly right-handed as determined by a modified version of the Edinburg Handedness Inventory (Oldfield, 1971), and had normal or corrected-to-normal-vision. This research was approved by the Psychology/Sociology Human Research Ethics Board (PSREB) at the University of Manitoba.

#### Stimulus and Procedure

**FIGURE 1:**
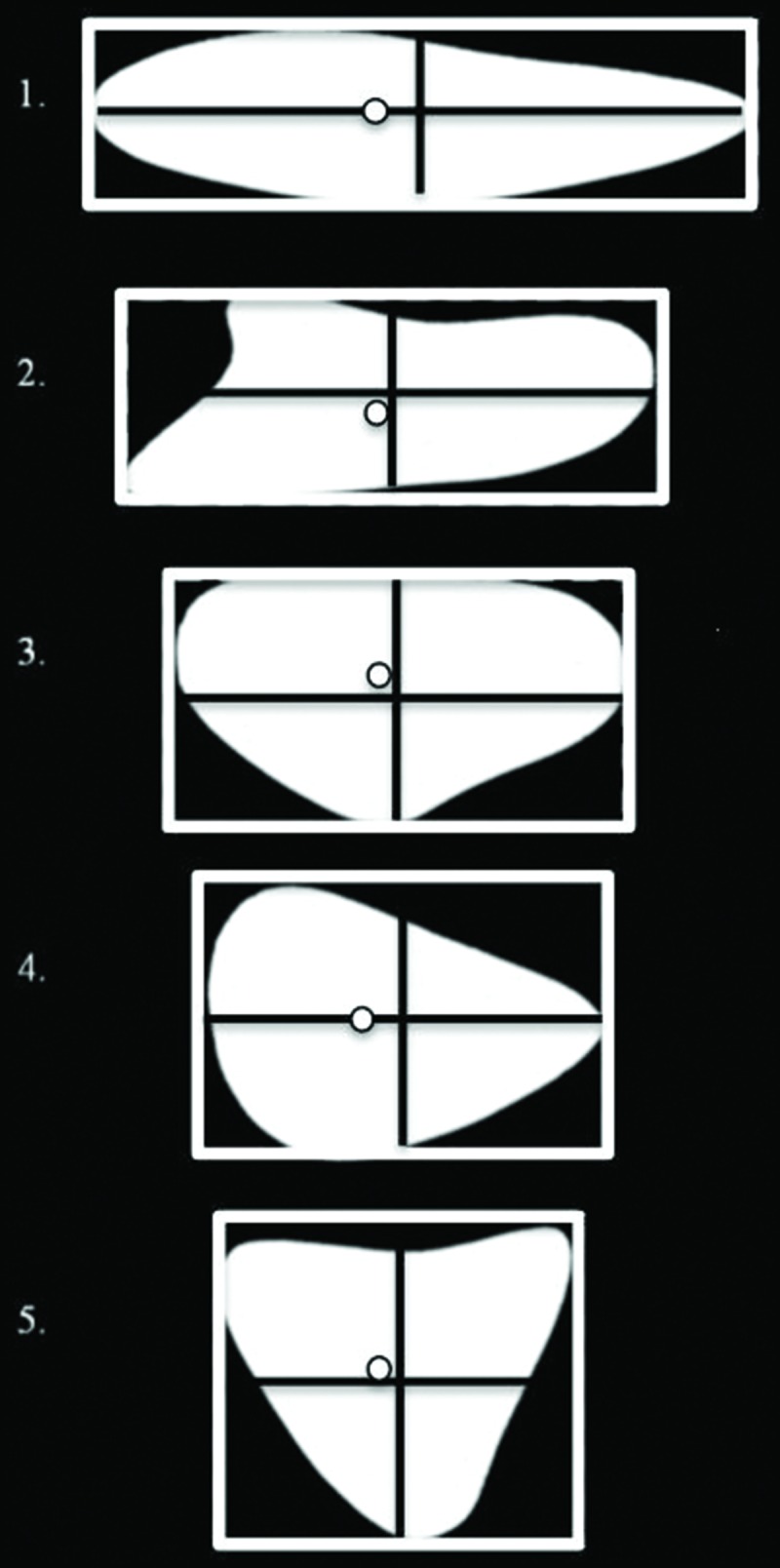
**Displays the Efron Shapes (dashed lines) and Blake shapes used in Experiment 1.** The Blake shapes in this Figure demonstrate COML objects. Maximum horizontal and vertical object dimensions are as follows: (1) 15.2 cm × 4.2 cm, (2) 12.2 cm × 5.2 cm, (3) 10.2 cm × 6.2 cm, (4) 9.0 cm × 7.1 cm, (5) 8.0 cm × 8.0cm. The COM location for each asymmetrical object is represented by a white circle on the object, with the following X and Y distances relative to the center of the object which is represented by the intersection of the black lines (1) 1 cm, 0 cm; (2) 0.3 cm, -0.5 cm; (3) 0.03 cm, 0.05 cm; (4) 1 cm, 0 cm; (5) 0.5 cm, 0.3 cm.

Participants were instructed to reach out “quickly but naturally” with their index finger and thumb and pick up randomly inter-mixed symmetrical (Efron blocks; Efron, 1968) and asymmetrical (Blake shapes; Blake, 1992) objects (all blocks weighed approximately 10 g). The Efron blocks differed in shape but were equal in surface area, and had the following horizontal and vertical dimensions: (1) 15.2 cm × 4.2 cm, (2) 12.2 cm × 5.2 cm, (3) 10.2 cm × 6.2 cm, (4) 9.0 cm × 7.1 cm, and (5) 8.0 cm × 8.0 cm (see **Figure 1**). The Blake shapes had smoothly bounded contours and lacked clear symmetry (see **Figure 1**). Thus, the positioning of stable grasp locations on asymmetrical Blake shapes requires the analysis of the entire shape (Goodale et al., 1994). Each of the five Efron blocks were matched with Blake shapes with identical maximum vertical and horizontal dimensions. In addition, each Blake shape was either presented with the COM oriented to the left or right of the object’s horizontal midline (objects were the mirror image of themselves), resulting in two asymmetrical object groups: Blake shapes with their COM shifted to the left (COML), and Blake shapes with their COM shifted to the right (COMR; see **Figure 1** for COM positions), and a third symmetrical group of Efron blocks.

Reach-to-grasp movements were recorded with an Optotrak Certus 3-D recording system (150 Hz sampling rate, spatial accuracy up to 0.01 mm; Northern Digital, Waterloo, ON, Canada). Two IREDs were fastened onto the participants’ index finger (positioned on the left side of the cuticle), thumb (positioned on the right side of the cuticle), and wrist (positioned on the radial portion of the wrist) of their right hand. An Eye-link II (250 Hz sampling rate, spatial resolution <0.5°; SR Research Ltd., Osgoode, ON, Canada) was used to record eye-movements in both tasks. Kinematic information from both the Optotrak and the Eyelink II was integrated into a common frame of reference via MotionMonitor software (Innovative Sports technology, Chicago, IL, USA). The Motion Monitor system integrates eye, head, and hand data in a common frame of reference.

Both eyes were calibrated using a nine point calibration/validation procedure on the computer monitor, after which, a black display board was fastened over the calibration area. The asymmetrical and symmetrical objects were suspended via small magnets to this display during the grasping paradigm. To ensure accurate calibrations of less than 1° error and reliability of binocular eye data, accuracy checks both immediately following calibration and after the completion of the experiment were taken by having participants fixate a marker on the display while positional eye data was obtained.

At the start of each trial, participants held their right hand stationary on the start button with their index finger and thumb together and their eyes closed. The experimenter signaled the beginning of each trial with verbal instructions for the participant to open their eyes. At that point, participants reached out as quickly, but as naturally as they could, and grabbed the object with their index finger and thumb (objects were positioned 30 cm from the start button), and placed it on the table in front of them. Each grasp was positioned vertically, such that participant’s index finger made contact with the object’s top edge, and their thumb made contact along the bottom edge. After the completion of the trial, participants returned to the starting position with their index finger and thumb on the start button and their eyes closed, until given the verbal command to start the next trial.

The shapes were always presented with their longest axis on the horizontal plane such that, for the asymmetrical objects, their COM was oriented to the left or right of the actual center of the object on any given trial. For the purpose of these experiments, the ‘center’ of an object corresponds to the horizontal and vertical midpoint of each object, the halfway point calculated from the maximum horizontal and vertical dimensions of the block. The COM refers to the point where all of the mass of the object is concentrated, based on the averaging of the surface area of the objects (see **Figure 1**). Each object was suspended in such a manner that every object’s vertical and horizontal center was aligned with the board’s center. All horizontal (X-axis) and vertical (Y-axis) coordinates were calculated from this location. For example, negative fixation or grasp locations represented locations to the left of or below the object’s horizontal and vertical center, respectively. To protect from false start or end times, the beginning of all trials started when wrist velocity reached 5 cm/sec and ended when wrist velocity decreased to 10 cm/sec. Each object was randomly presented five times, for a total of 75 experimental trials. Sessions took ∼ 1 h to complete.

#### Data Analysis

The main goal of Experiment 1 was to investigate where participants looked when grasping asymmetrical shapes, when compared to symmetrical shapes, and whether fixations were linked to grasp locations. Thus, we were mainly concerned with fixation and grasp locations on the objects, and how these locations differed between conditions (symmetrical Efron blocks vs. asymmetrical Blake shapes (COML, COMR)). For the purpose of clarification in the results, object shapes are referred to individually by object type and size [COML(1-5), COMR(1-5), symmetrical shapes(1-5)] or collectively based on object category (collapsed across all sizes; COML, COMR, and symmetrical shapes).

Fixation locations (both X and Y positions) and durations were determined by a dispersion algorithm (see Salvucci and Goldberg, 2000), with a minimum duration threshold of 150 ms and a maximum dispersion threshold of 1 cm. The dispersion algorithm identifies fixations from the raw eye position data points when consecutive data points are located within a specified spatial window (maximum dispersion threshold) for a minimum period of time (minimum duration threshold). Fixations were calculated from the point when participants first opened their eyes until they made contact with the object. All X and Y coordinates of the gaze were relative to the center of the object.

For all analyses, significance levels of *p* < 0.05 were used. Analyses were carried out on the mean values computed across repeated trials in a given condition. For any main effects or interactions, Bonferroni adjusted planned comparisons were carried out. To explore differences between fixation locations (first and second fixations) across object types (COML, COMR, and symmetrical objects), and the five object sizes (X and Y dimensions: (1) 15.2 cm × 4.2 cm, (2) 12.2 cm × 5.2 cm, (3) 10.2 cm × 6.2 cm, (4) 9.0 cm × 7.1 cm, and (5) 8.0 cm × 8.0 cm; see **Figure 1**), a 2 fixation × 3 object type × 5 object size repeated measure analysis of variance (rmANOVA) was carried out for fixation positions along the X- and Y- axes separately. To explore whether fixation locations were linked to grasp axis locations for each object type, a 2 location (first fixation location by grasp axis location) × 5 object size rmANOVA was carried out for each object type (COML, COMR, and symmetrical objects) relative to X-axis locations.

For the kinematic data, rmANOVA’s, with within subject variables of object type and object size, were carried out on the following dependent variables: grasp line, maximum grip aperture between the index finger and thumb (MGA), time to MGA, peak wrist velocity, and reach duration A participant’s ‘grasp line’ location, an imaginary line connecting the contact points of the index finger and thumb on the object, was calculated by determining the perpendicular distance between the ‘grasp line’ and the horizontal midpoint of the object.

### Results

#### Fixation Data

**FIGURE 2:**
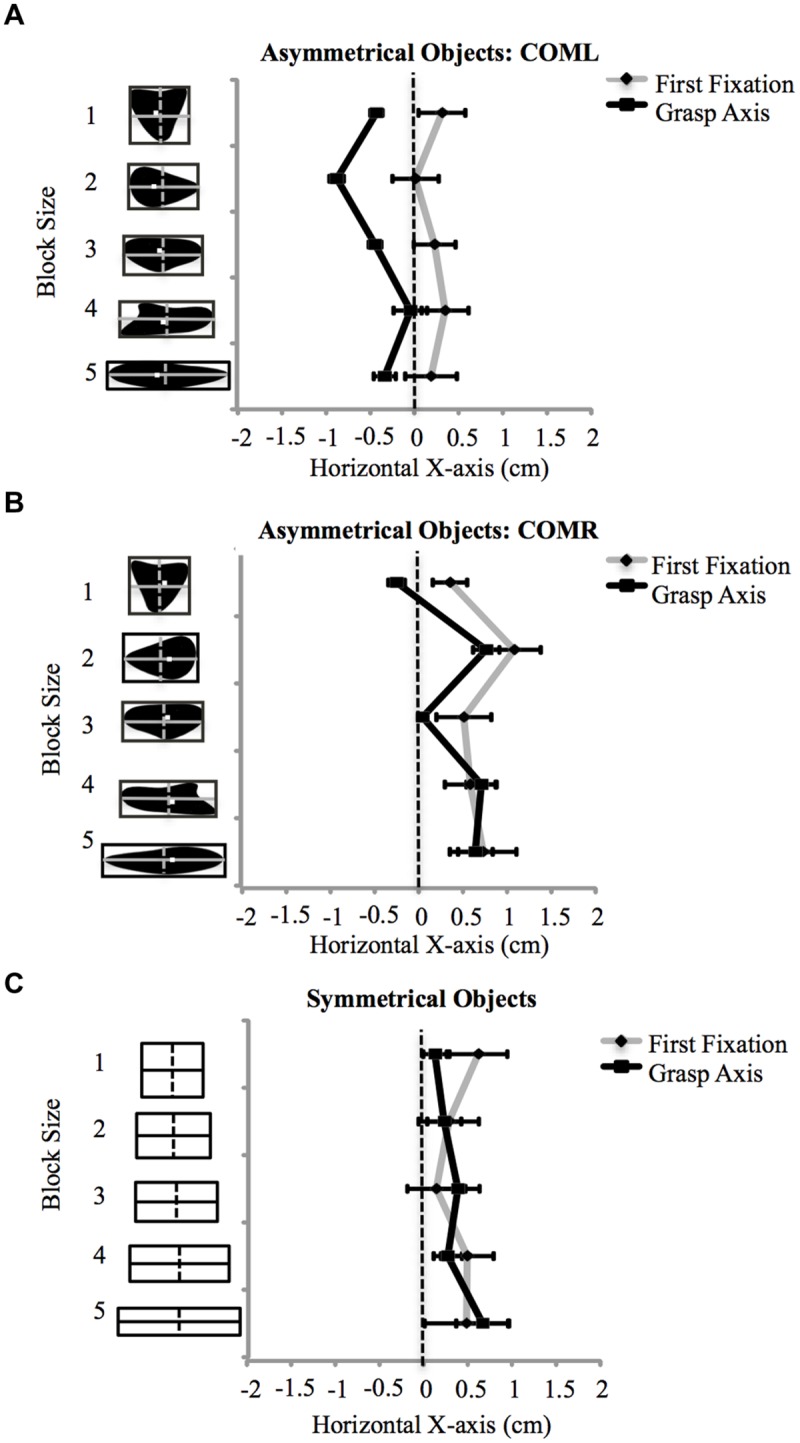
**Displays first fixation and grasp axis locations along the X-axis (cm) across object size for each object group separately in Experiment 1: **(A)** COML, **(B)** COMR, **(C)** symmetrical objects.** Negative values represent fixation locations to the left of the object’s horizontal center. Error bars represent the standard error of the mean. The COM location for each asymmetrical object is represented by a white square on the object.

A first fixation was detected in 97% of all experimental trials. Ninety percent of those trials contained more than one fixation. Fixations were not detected in 3% of trials due to loss of eye data (e.g., loss of corneal reflection or IRED interference resulted in loss of data or inaccurate fixation points outside of the calibrated region), and these trials were excluded from any further analyses. A 2 fixation × 3 object type × 5 object size rmANOVA revealed the expected influence of COM location on fixation locations (i.e., fixations were drawn toward the COM location) along the X-axis [*F*(2,26) = 12.46, *p* < 0.001]. That is, fixation locations were significantly more to the left (*M* = 0.13 cm to the right of the center, *SE* = 0.23) when grasping the COML objects when compared to the COMR objects (M = 0.68 cm to the right of the center, SE = 0.25; *p* < 0.001). No differences in fixation locations to symmetrical objects were observed (*M* = 0.33 cm to the right of the object’s center, *SE* = 0.33) when compared to asymmetrical fixation locations. No significant differences between first and second fixation locations along the X-axis were apparent in any object category (COML, COMR, symmetrical objects). A main effect of object type showed some variability in fixation locations within the different object categories [*F*(8,104) = 6.20, *p* < 0.001]. For the COML objects, fixation locations were significantly more to the left when grasping COML 4 when compared to COML 3 (see **Figure 2A**). For the COMR objects, fixation locations were significantly more to the right when grasping COMR 4 when compared to COMR 2, 3, and 5 objects (see **Figure 2B**). No significant differences in fixation locations were observed across objects within the symmetrical object category sizes (see **Figure 2C**).

**FIGURE 3:**
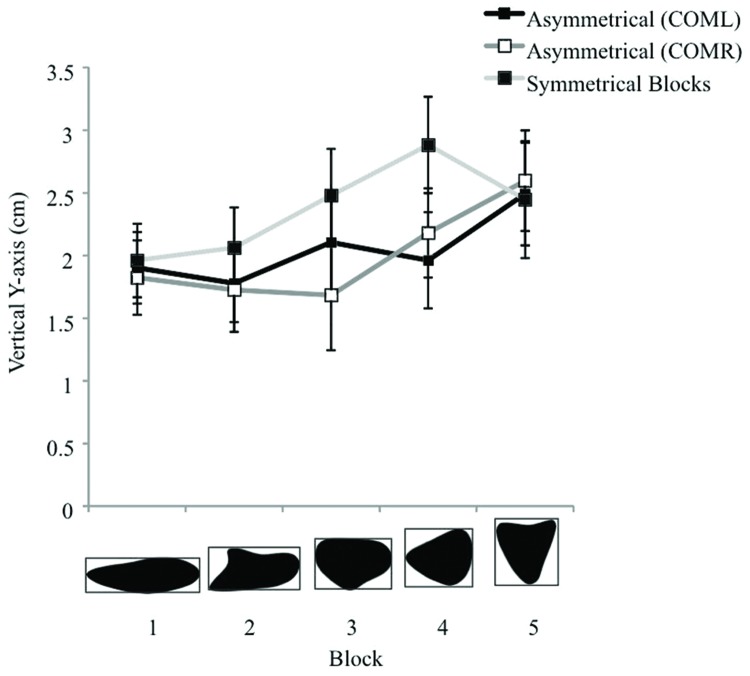
**Displays the vertical fixation locations (cm) when grasping the Asymmetrical (COML, COMR) and symmetrical objects in Experiment 1.** Positive values represent fixation locations above the object’s vertical center. Error bars represent the standard error of the mean.

Along the Y axis, a 2 fixation × 3 object type × 5 object size rmANOVA revealed a significant main effect of object size [*F*(4,52) = 8.82, *p* < 0.001]. In general, as object size increased in height, fixation locations moved progressively higher. *Post hoc* comparisons showed these differences to be significant between the tallest object categories (5: *M* = 2.51 cm, *SE* = 0.40) and 4: *M* = 2.34 cm, *SE* = 0.36) with the two shortest object categories (2: *M* = 1.86 cm, *SE* = 0.31 and 1: *M* = 1.89 cm, *SE* = 0.28; *p*’s < 0.05). A significant main effect of object type (COML, COMR, symmetrical objects) [*F*(2,26) = 9.55, *p* = 0.001] showed differences in the Y-axis locations to asymmetrical objects relative to symmetrical shapes. Fixation locations to both asymmetrical COML and COMR objects were significantly lower on the objects (*M*’s = 2.05 cm (*SE* = 0.34) and 2.00 cm (*SE* = 0.35) above the center, respectively) compared to fixations to symmetrical objects (*M* = 2.37 cm above center, *SE* = 0.34). A significant object type by object size interaction [*F*(8,104) = 3.23, *p* = 0.02] showed that Y fixation locations were located significantly higher when grasping symmetrical shapes 3 and 4, than when grasping COMR 3, COML 4, or COMR 4 objects (*p*’s < 0.05; see **Figure 3**).

#### Kinematic Data

Of all experimental trials, 4% were removed due to loss of IRED signal from the camera (due to obstruction). Results again showed the expected influence of COM location on grasp location (i.e., grasp locations were drawn to COM location). A 3 (object type) by 5 (object size) rmANOVA showed a significant main effect of object type (COML, COMR, symmetrical objects) [*F*(2,26) = 28.74, *p* < 0.001]. Grasp axis locations for COML objects were significantly more to the left (*M* = 0.43 cm to the left of the object’s center, *SE* = 0.06) when compared to COMR and symmetrical objects (*p*’s < 0.05; *M*’s = 0.41 cm (*SE* = 0.09) and 0.29 cm (*SE* = 0.19) to the right of the object’s center, respectively). No significant differences were observed between the symmetrical and COMR objects.

An object type by object size interaction [*F*(8,104) = 11.03, *p* < 0.001] showed differences in grasp axis locations within each object category. For COML objects, grasp axis locations were significantly more to the left when grasping COML four when compared to all other objects (*p*’s < 0.05; see **Figure 2A**). For COMR objects, grasp axis locations were significantly more to the left when grasping COMR 5 (the shortest in width) when compared to COMRs 4, 2, and 1 (*p*’s < 0.05). In addition, grasp axis locations for COMR 3 were positioned significantly more to the left of grasp axis locations to COMRs 2 and 4 (*p*’s < 0.05; see **Figure 2B**). For the symmetrical objects, no differences in grasp axis locations were observed (see **Figure 2C**).

A 3 (object type) by 5 (object size) rmANOVA on MGA showed a significant main effect of object size [*F*(4,52) = 121.70, *p* < 0.001]. Across object categories, MGA increased with object height. Planned *post hoc* comparisons revealed significant increases in MGA between all object sizes except object size 3 with 4 (*p*’s < 0.05). On average, participants obtained MGA 75% through the reach-to-grasp movement. Across objects, no significant main effects or interactions were observed for peak velocity or total reach time (*p*’s > 0.05). On average, participants’ peak velocity was 113 cm/sec (*SE* = 5) and their completed reach-to-grasp movement was 594 ms (*SE* = 38).

#### Fixation Locations (X-axis) vs. Grasp Axis Locations

A 2 location (first fixation location by grasp axis location) × 5 object size rmANOVA revealed that overall, fixation locations supported grasp axis locations for all object categories except COML objects [*F*(1,13) = 8.90, *p* = 0.01]. *Post hoc* comparisons revealed that across objects, grasp axis locations to COML objects were significantly more to the left of first fixation locations on the same objects (*p*’s < 0.05; see **Figure 2A**). A location by object size interaction was observed for COMR objects [*F*(4,52) = 3.38, *p* = 0.02]. *Post hoc* analysis revealed that fixation locations supported grasp locations for all objects except COMR 5, where first fixations were significantly more to the right of grasp axis locations (*p*’s < 0.05; see **Figure 2B**). Finally, a significant location by object size interaction was observed for the symmetrical objects [*F*(4,52) = 4.62, *p* = 0.003], however, *post hoc* comparisons did not reveal any significant differences in first fixation and grasp axis locations across these objects (*p’s* > 0.05; see **Figure 2C**).

## Experiment 2

The results from Experiment 1 demonstrate grasp and fixation locations were influenced by COM location. Grasp and fixation locations to asymmetrical objects with their COM to the left of the object’s midline were found to be more to the left of grasp and fixation locations to asymmetrical objects whose COM was to the right of the object’s midline, grasp and fixation locations for symmetrical objects were in between. In all instances, there were no differences in grasp axis locations and fixation locations along the X-axis, except when grasping the COML objects and the smallest width asymmetrical (COMR 5) object. This experiment showed both a dissociation in fixation locations and grasp locations on asymmetrical objects due to COM position as well as a tendency to fixate areas closer to an object’s COM when grasping asymmetrical vs. symmetrical shapes. However, despite COM location exerting a large effect on where we looked and grasped objects, we are still unsure how systematic changes in COM location (i.e., an increase in distance of the COM of an object from its horizontal center) would influence visuomotor control. In Experiment 2, we wanted to further explore the relationship between COM location and visuomotor control by investigating how fixation and grasp locations are affected by changes in COM distance. In other words, will fixation and grasp positions be influenced by systematic changes in COM distance from an object’s midline? To explore this, the COM of three different objects were dissociated from each object’s horizontal midline at three different distances and fixation and grasp locations were recorded. It was expected that participant’s grasp axis and fixation locations would be shifted away from the horizontal center of the objects, towards the object’s COM, and this distance would increase with increases in COM locations from that point.

### Materials and Methods

#### Participants

Fifteen undergraduate psychology students (11 female) between the ages of 18 and 32 (*M* = 20 years-old) were recruited for participation in this study. All participants were right-handed as determined by a modified version of the Edinburg handedness inventory (Oldfield, 1971) and had normal or corrected-to-normal-vision. This research was approved by the PSREB at the University of Manitoba.

#### Stimulus and Procedure

**FIGURE 4:**
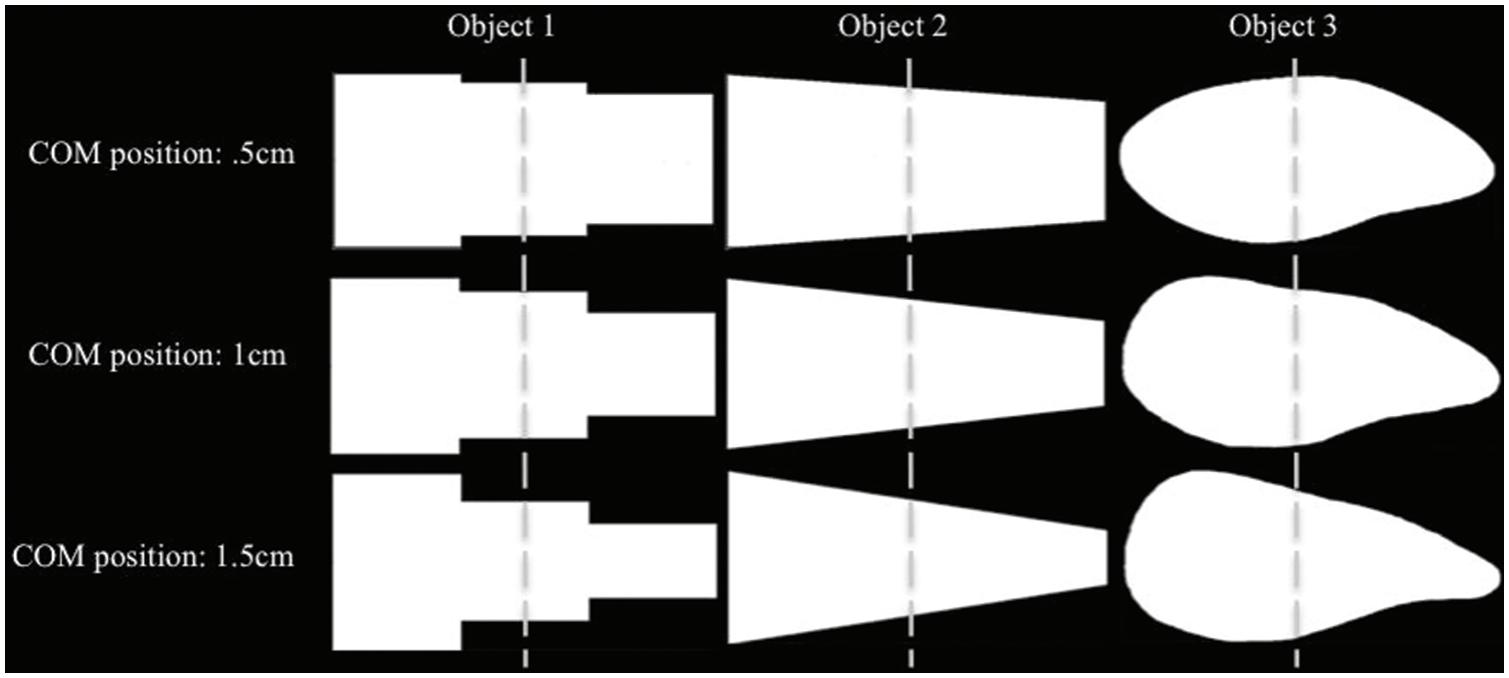
**Displays the two symmetrical and one asymmetrical object used in Experiment 2.** Each object shape was manipulated such that the COM of each object was located at three distances from the object’s horizontal center: 0.5 cm, 1 cm, and 1.5 cm. The dashed line transects the object’s horizontal center.

All equipment, procedures, and instructions were identical as that described in Experiment 1. The stimuli used in this task were one asymmetrical object (modeled after the Blake shapes) and two differently shaped symmetrical objects with one axis of symmetry. These three distinct objects were selected to explore the effects that COM distance, across a variety of shapes, exerted on grasp and fixation selection. Each object was presented in three variations, with the COM at 0.5 cm, 1 cm, and 1.5 cm from the center of the object (total of nine objects; due to changes in COM, slight variations in shape within object categories occur; see **Figure 4**). Each object was presented 16 times [eight presentations with the COM oriented to the left (COML) and eight presentations with the COM oriented to the right (COMR) of the subjects midline], for a total of 144 trials. Sessions took approximately one and a half hours to complete.

#### Analysis

To explore the immediate influence of COM position on fixation locations, only first fixations were analyzed in this experiment. To investigate whether changes in COM location were affecting grasp axis locations and gaze fixation locations along the X- and Y- axis, rmANOVA’s, with factors of COM position (COML, COMR), COM distance (COM 0.5 cm, 1 cm, and 1.5 cm away from horizontal center), and object type (three different objects: object 1, 2, and 3), were performed on all dependent measures. To explore whether fixation locations supported grasp axis locations for each object type across changes in COM distance and COM position, a 2 location (first fixation location X-axis, grasp axis location) × 3 object type × 3 COM distance × 2 COM position rmANOVA was carried out.

### Results

#### Fixations

On average participants made 2.03 fixations per trial. In 98% of all trials a first fixation was detected. Fixations were not detected in 2% of trials due to loss of eye data (e.g., loss of corneal reflection or IRED interference), and these trials were excluded from any further analyses.

Object type exerted a significant main effect on both X [*F*(2,28) = 3.86, *p* = 0.03] and Y [*F*(2,28) = 4.42, *p* = 0.02] fixation locations. While fixation locations to object 1 were positioned more to the right (*M* = 0.61 cm to the right of the object’s midline, *SE* = 0.25) of fixations to object 2 and 3 (*M*’s = 0.42 cm (*SE* = 0.27) and 0.44 cm (*SE* = 0.23) to the right of the object’s midline, respectively), *post hoc* analysis only showed significant differences along the vertical axis; first fixations to object 3 (*M* = 1.77 cm above the object’s center, *SE* = 0.36) were significantly higher when compared to object 2 (*M* = 1.54 cm above the object’s center, *SE* = 0.32; *p* < 0.05). First fixations to object 1 were located 1.71 cm (*SE* = 0.36) above the object’s center.

**FIGURE 5:**
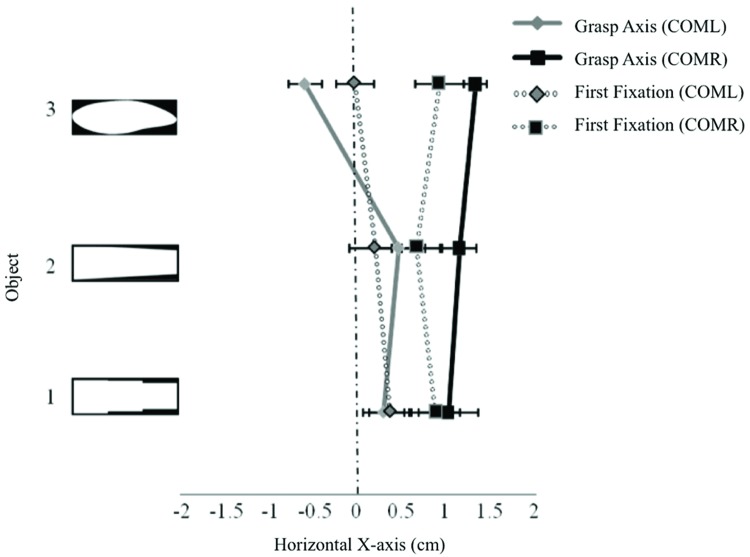
**Shows the mean first fixation and grasp axis locations along the horizontal X-axis collapsed across COM distance for the three different object types in Experiment 2.** Negative values represent positions that are located to the left of the object’s horizontal midline. Error bars represent the standard error of the mean.

Expected differences in fixation locations based on COM position (left vs. right) along the X-axis were observed [*F*(1,14) = 21.95, *p* < 0.001]. First fixations were significantly more to the left (*M* = 0.17 cm to the right of the object’s horizontal midline, *SE* = 0.24) for COML objects when compared to COMR objects (*M* = 0.81 cm to the right of the midline, *SE* = 0.27). An object type by COM position interaction [*F*(2,28) = 4.49, *p* = 0.02] showed that fixations were significantly more to the left for object 3 compared to object 1 for COML objects; for COMR objects, fixations to object 3 were significantly more to the right when compared to object 2 (*p’*s < 0.05; see **Figure 5**).

**FIGURE 6:**
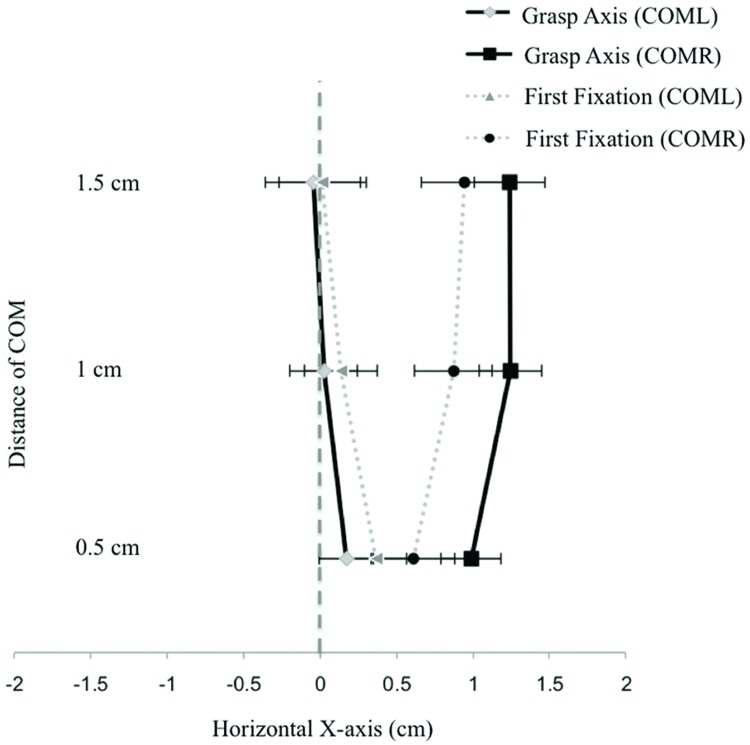
**Displays first fixation positions and grasp axis locations across the three COM distances (collapsed across object) for both leftward and rightward oriented COMs in Experiment 2.** Negative values represent positions that are located to the left of the object’s horizontal midline. Error bars represent the standard error of the mean.

Center of mass distance also influenced fixation locations. A COM distance by COM position interaction for the X-axis fixation locations [*F*(2,28) = 12.28, *p* < 0.001] revealed that fixation locations in the COML condition moved increasingly leftward as the COM moved increasingly left (note: participants started with a rightward bias; see **Figure 6**). Significant differences were observed between COM distance.5 cm with COM distance 1 cm and 1.5 cm (*p’*s < 0.05). For COMR objects, fixation locations moved increasingly rightward as the COM moved increasingly right. Significant differences were observed between COM distance.5 cm with COM distance 1 cm and 1.5 cm (*p’*s < 0.05; see **Figure 6**). Along the Y axis, a main effect of COM distance [*F*(2,28) = 3.90, *p* = 0.03] showed that as COM locations were positioned further from the center (resulting in decreases in object height at the center of the objects) fixation locations moved closer to the object’s vertical center [*M*’s = 1.80 cm (*SE* = 0.36), 1.65 cm (*SE* = 0.34), and 1.58 cm (*SE* = 0.33), respectively]. Significant differences were observed between the tallest object size (COM distance of 0.5 cm) and the shortest object size (COM distance of 1.5 cm; *p* < 0.05).

#### Grasp Location

Object shape had a significant effect on grasp location [*F*(2,28) = 7.33, *p* = 0.003], participants’ grasp axis locations were significantly farther away from the object’s center for object 2 (*M* = 0.80 cm to the right of the object’s midline, *SE* = 0.21) compared to object 3 (*M* = 0.36 cm to the right of the object’s midline, *SE* = 0.14; *p* < 0.05). A significant main effect of COM position [*F*(1,14) = 30.88, *p* < 0.001] and an object by COM position interaction [*F*(2,28) = 13.53, *p* < 0.001] were also observed. Grasp axis locations were significantly more to the left for COML objects (*M* = 0.05 cm to the right of the midline, *SE* = 0.23) when compared to COMR objects (*M* = 1.16 cm to the right of the midline, *SE* = 0.20). The object by COM position interaction showed no differences between objects in grasp axis locations when the objects were oriented to the right of the center (see **Figure 5**). With COML objects, grasp axis locations for object 3 were significantly more to the left than grasp axis locations for objects 1 and 2 (*p’*s < 0.05; see **Figure 5**). No significant main effects of COM distance or any object or COM position by COM distance interactions were observed (*p’s* > 0.05).

#### First Fixation Location (X-axis) vs. Grasp Axis Location

Results revealed a significant COM location by object type by COM position three-way interaction [*F*(2,28) = 6.50, *p* = 0.01]. *Post hoc* analysis showed that first fixations and grasp axis locations (collapsed across COM distance) for all objects in both COM positions (COML, COMR) were the same, except for object 3. When object 3’s COM was oriented to the left of the center, fixation locations were found to be positioned significantly more to the right of grasp axis locations (see **Figure 5**).

### Discussion

The characteristics of reaching and grasping objects have been well documented (e.g., Jeannerod, 1986; Gentilucci et al., 1991; Jakobson and Goodale, 1991; Paulignan et al., 1991; Galletti et al., 2003; Castiello, 2005). Traditionally, however, the primary concern in the reaching and grasping literature has been with how the opening of the hand is coordinated with the hand’s approach towards the target objects, typically using regular shaped objects or objects where the grasp points were controlled. Few studies have examined the selection of grasp locations when grasping irregularly shaped objects and to our knowledge, no studies have examined the selection of grasp and gaze behaviors during various manipulations of object COM. The purpose of this research was to examine the variability in fixation locations across several conditions on irregular asymmetrical objects and explore the relationship of these fixations to grasp locations on those same objects. In Experiment 1, we investigated fixation and grasp locations to contoured objects that had an asymmetrical design and compared this behavior to grasps made to symmetrical objects that had identical maximum horizontal and vertical dimensions. In Experiment 2, we explored the effects of object shape and COM location on fixation and grasp locations. The combined results from these studies demonstrate several significant effects of object properties on grasp and fixation locations, including: COM position (COML vs. COMR) influences where we grasp and where we look when picking up an object (Experiments 1 and 2); fixations are less linked to grasp locations when we are grasping asymmetrical objects with the COM oriented to the left of the object’s midline (Experiments 1 and 2); object irregularity results in more central fixations (Experiment 1), and; increasing COM distance from the objects horizontal midline affects grasp and fixation locations differently (Experiment 2). These findings will be discussed in turn.

Results from Experiments 1 and 2 demonstrated that both fixation and grasp locations were influenced by COM location (COML vs. COMR). That is, grasp and fixation locations were drawn towards COM location, resulting in positional differences in visuomotor control between leftward and rightward oriented COM positions. This manipulation, however, also systematically changed other factors of the objects, which arguably could have influenced grasp and fixation behavior as well. For example, the side of the object where the COM was located also appears larger than the other side. This difference in object size could have biased the perception of the ease of grasp and influenced results. Despite this caveat, COM position, rather than differences in object width, does seem to be the determining factor mediating grasp and fixation locations, consistent with previous reaching and grasping findings (e.g., Jeannerod, 1988; Goodale et al., 1994; Lederman and Wing, 2003; Marotta et al., 2003; Kleinholdermann et al., 2007). For example, in Experiment 1, for asymmetrical objects 1 and 3, the center of the object is approximately the same width as at the COM location. However, we still see a bias in grasp location depending on the position of the COM (Left vs. Right). If object width was influencing grasp positions due to ease of grasp, then we would not expect such a large influence of COM position with these objects as the center would be just as likely to be grasped in both COM orientations. Additionally, in Experiment 2 (for objects 1 and 2), if participants were biased to grasp the larger parts of the objects we would expect to see grasp locations much more influenced by these areas. Rather, we saw grasp locations much closer to the object’s COM despite very large widths present on one side (especially object 1). Together these finding advance on the existing literature by including the link between fixation and grasping behavior to irregular shaped asymmetrical objects and highlight the importance of COM position for both behaviors. This influence was apparent in both studies, despite small deviations in COM position (within 1.5 cm) from the blocks midline.

The manipulation of COM location also revealed several interesting differences in eye-hand behavior. For instance, fixation and grasp locations for COMR objects, while more to the right, were not found to be significantly different in position than those to symmetrical shapes. Potentially, this lack of difference is due to the slight rightward grasp and fixation biases when picking up symmetrical objects – results demonstrated in previous studies (Desanghere and Marotta, 2011; Prime and Marotta, 2013). Additionally, in both instances overall fixation locations were linked to grasp location as demonstrated in previous studies (de Grave et al., 2008; Brouwer et al., 2009; Desanghere and Marotta, 2011; Prime and Marotta, 2013; Bulloch et al., 2015). When the COM of the asymmetrical objects were positioned to the left of the horizontal midline (COML), despite grasp positions that were to the left of the center of the objects, fixations were again found to be in close proximity to fixations when grasping symmetrical shapes (i.e., to the right of the object’s midline); but still significantly more to the left than fixations to COMR objects, thus demonstrating a COM position influence on where we look. In this condition, these rightward fixation locations did not support grasp locations as with symmetrical and COMR objects. These results suggest a rightward fixation bias when interacting with both symmetrical and asymmetrical shapes, regardless of the position of the object’s COM. Rightward fixation biases have been shown in other studies when participants are instructed to look at the mid point of an object. For example, previous research has shown that when participants “visually bisect” complex stimuli, the subjective midpoint is placed to the right of the object’s true center (Elias et al., 2005; Rhode and Elias, 2007). In addition, Handy et al. (2003) showed that visual spatial attention was drawn to graspable objects (tools) in the right visual hemifield. These results suggest visual field asymmetries in the processing of action-related attributes and spatial attention, with attention to specific object features aiding in recognition of the motor affordance (Handy et al., 2003).

Interestingly, recent research has demonstrated an effect of handedness on grasp point selection when picking up symmetrical objects (Paulun et al., 2014). Consistent with previous research (e.g., Desanghere and Marotta, 2011; Prime and Marotta, 2013), Paulun et al. (2014) demonstrated a slight rightward grasp bias relative to the object’s COM, when participants were picking up symmetrical objects. Conversely, a slight leftward grasp bias was demonstrated when participants grasped the objects with their left hand. These authors suggest that the variation in grasp point selection is the result of a compromise between obtaining maximum stability (grasp points near the COM) and a slight lateral deviation toward the side of the grasping hand, potentially to increase the visibility of the object as a whole while lifting it (Paulun et al., 2014). Whether fixations would support this leftward grasp bias when grasping with the left hand, or whether fixations would be drawn to the right side of the object as demonstrated in the present experiments is yet to be determined. Indeed, the results from the present study do suggest a persistent rightward visuospatial bias, despite grasp locations to the left of an objects midline when the COM of the object is oriented in that direction. For example, and as previously mentioned, fixations to COML objects in Experiment 1 did not coincide with where participants were grasping the objects. Grasp locations were drawn to COM position (positioned to the left of the horizontal midline); however, a dissociation between fixation locations and grasp positions were observed in this condition, with fixation locations to the right of the objects center. This effect was also demonstrated in Experiment 2, where a rightward fixation bias (relative to the center of the object) and a difference in this position relative to grasp locations for COML objects (object 3) were also shown. Overall, these results are similar to Prime and Marotta (2013) who demonstrated a decoupling in fixation locations with grasp locations when performing a memory guided grasping task to symmetrical shapes. They conclude that the purpose of initial fixations for the purpose of memory guided grasping is to provide the visuomotor system with a general perceptual analysis of the blocks properties. The present results suggest that visual attention and grasping movements become loosely coupled in some conditions, with grasp locations largely mediated by COM location and fixations only supporting grasp locations when they are to the right of the object’s horizontal midline. When grasp locations are to the left of the object’s midline, fixations are drawn to the right of center; similar to findings during perceptual tasks.

In addition to this slight rightward bias, our results also demonstrated that more irregular structures are eliciting fixation locations that are more central on the objects (lower on the objects when compared to fixations to symmetrical shapes; Experiment 1), regardless of COM position. Overall, object irregularity is resulting in more centralized fixation locations on the objects, perhaps to maintain maximum visibility of the object’s shape, where placement of both index finger and thumb is important for grasp stability on the irregular shaped objects. Indeed, allocating attention to a specific location on an object, in this case a more central location, has been shown to result in faster and more accurate processing of form information in regions of space surrounding that location (Bashinski and Bacharach, 1980; Hoffman and Nelson, 1981; Downing, 1988). This more central view would provide a more holistic representation during grasping, taking the whole object into consideration. Definitely, when we reach out to pick up an object, we are able to do this with great precision, regardless of the object contours. To do this, information about intrinsic (e.g., size, shape) and extrinsic (e.g., distance, orientation) features need to be transformed in order to develop a motor plan for movement execution (Jeannerod, 1988). Our results suggest that the visual analysis of complex objects is different from that of symmetrical shapes. While research supports that when grasping simple objects, fixation locations are tightly linked to index finger grasp location, the more complex structures in these experiments are eliciting fixation locations that are more central on the objects (lower on the objects when compared to fixations to symmetrical shapes) and close to the horizontal midpoint of the block regardless of COM position.

In Experiment 2 we also showed that increasing COM distance from the objects horizontal midline affects grasp and fixation locations differently. In this experiment, the influence of COM position on grasp and fixation locations were explored through manipulations of COM distance from the horizontal midline of the objects (up to 1.5 cm from the object’s horizontal center). These manipulations did not have the expected systematic influence on grasp point selection. Consistent with Experiment 1, changes in COM position affected both grasp and gaze positions, however, increased distances from that point did not exert a further influence on grasp locations. Regardless of the COM position, and collapsed across all object types, significant increases in distance for grasp locations from the center of the objects were not observed with increased distances of COM. Unlike grasp locations, fixations were influenced by systematic changes in COM location, with fixations to objects with COM distances of 1 cm and 1.5 cm away from the center, significantly further away from fixation locations to the objects with the COM closest to the object’s center (0.5 cm) in both COM positions. Interestingly, a rightward fixation bias was again present for all objects. This research suggests that fixations are influenced by COM position and distance, but tend to still maintain a “central” position, with a slight rightward bias relative to the center of the object. The maintenance of these centralized fixation locations were also observed along the vertical axis as well, with fixation locations moving progressively closer to the objects center with systematic increases in COM position (resulting in a decrease in object height at the block’s midpoint). Again, supporting the notion that when grasping irregular shapes, a central view provides a more holistic representation that may be needed for monitoring both index and thumb placement on irregular objects.

Taken together, our findings not only highlight the importance of including irregular, non-symmetrical objects in visuomotor paradigms but also reveal how object features differentially influence gaze vs. grasping during object interaction. The importance in allocating visual attention to an object’s COM or the exact grasp location of the index finger on asymmetrical objects may become more important when interacting with heavier objects. When we reach out to pick up an object, the anticipated mass of that object automatically influences anticipatory grip forces (i.e., grip force is scaled proportional to the expected weight of an object, which is based on its size and type of material; Gordon et al., 1991). In other words, when we reach out to grasp an object using our index finger and thumb, the opposing digits exert a grip force (forces that are equal and opposite) to hold the object level. If the COM of the object is to one side of our grasp location, this offset position results in a turning force or torque. If we are to successfully grasp this object, we can either increase our grip force in order to generate the torsional friction needed to offset the rotation of the object around the grasp axis and keep the object level during the lift, or move our grasp axis to intersect the object’s COM (Wing and Lederman, 1998; Endo et al., 2011). Since the objects in this study were relatively lightweight, easily compensated for by increases in grip force, the placement of our grasp axis with varying degrees of COM changes becomes less important for grasp location selection. Despite this, however, it is apparent that we attend to changes in COM location, despite these locations not necessarily dictating our exact grasp location. While visual attention to asymmetrical objects remains relatively central (with a slight rightward bias), we are clearly sensitive to slight changes in COM location. Unlike grasping, in which changes in COM can be easily compensated for in grip force, the eyes have to attend to changes in relevant properties of the objects to help mediate this process. As you can imagine, the placement of our grasp would become progressively linked to an object’s COM if these changes also coincided with an increase in object mass and, if this were to occur, it would be important to be attending to these locations. Further research is needed to explore these factors as well as the persistent rightward visuospatial bias observed when grasping objects and the influence of handedness on gaze and grasp point selection during object manipulation.

## Conflict of Interest Statement

The authors declare that the research was conducted in the absence of any commercial or financial relationships that could be construed as a potential conflict of interest.
